# Functional RelBE-Family Toxin-Antitoxin Pairs Affect Biofilm Maturation and Intestine Colonization in *Vibrio cholerae*

**DOI:** 10.1371/journal.pone.0135696

**Published:** 2015-08-14

**Authors:** Yuning Wang, Hui Wang, Amanda J. Hay, Zengtao Zhong, Jun Zhu, Biao Kan

**Affiliations:** 1 Department of Microbiology, Nanjing Agricultural University, Nanjing, China; 2 State Key Laboratory for Infectious Disease Prevention and Control, National Institute for Communicable Disease Control and Prevention, Chinese Center for Disease Control and Prevention, Beijing, China; 3 Department of Microbiology, University of Pennsylvania Perelman School of Medicine, Philadelphia, Pennsylvania, United States of America; 4 Collaborative Innovation Center for Diagnosis and Treatment of Infectious Diseases, Hangzhou, China; University of Malaya, MALAYSIA

## Abstract

Toxin–antitoxin (TA) systems are small genetic elements that typically encode a stable toxin and its labile antitoxin. These cognate pairs are abundant in prokaryotes and have been shown to regulate various cellular functions. *Vibrio cholerae*, a human pathogen that is the causative agent of cholera, harbors at least thirteen TA loci. While functional HigBA, ParDE have been shown to stabilize plasmids and Phd/Doc to mediate cell death in *V*. *cholerae*, the function of seven RelBE-family TA systems is not understood. In this study we investigated the function of the RelBE TA systems in *V*. *cholerae* physiology and found that six of the seven *relBE* loci encoded functional toxins in *E*. *coli*. Deletion analyses of each *relBE* locus indicate that RelBE systems are involved in biofilm formation and reactive oxygen species (ROS) resistance. Interestingly, all seven *relBE* loci are induced under the standard virulence induction conditions and two of the *relBE* mutants displayed a colonization defect, which was not due to an effect on virulence gene expression. Although further studies are needed to characterize the mechanism of action, our study reveals that RelBE systems are important for *V*. *cholerae* physiology.

## Introduction

*Vibrio cholerae* is a gram-negative bacterium and the etiological agent of the pandemic disease cholera. While *V*. *cholerae* colonizes the intestine of its human host following ingestion of contaminated food and water, it is also found in the aquatic environment. In order to transition to an infective state in the host, *V*. *cholerae* senses host conditions and induces its virulence program. The result of this virulence program is production of two major virulence factors: cholera toxin (CT) and a toxin co-regulated pilus (TCP). CT is a potent enterotoxin that can lead to severe watery diarrhea and acute dehydration [1,2]. TCP is a type IV pilus that is critical for colonization of the host intestine [3]. The *tcp* cassette resides in the Vibrio Pathogenicity Island (VPI). It consists of the pilus biogenesis genes as well as structural protein genes, including the major pilin subunit TcpA [4]. In order to survive in both aquatic and host environments, *V*. *cholerae* has the ability to cope with harsh conditions during the transition to and growth within the host environment. It can form biofilms that are important for environmental survival [5]. It is also resistant to different stressors such as oxidative compounds and bile [6,7]. Bile is a host digestive secretion consisting of a complex mixture of lipids, proteins, bile salts, and other compounds. It has antimicrobial activity, and certain components can also act as a host signal to modulate *V*. *cholerae* colonization and virulence factor expression[8]. For other bacteria, environmental stressors such as these can promote transcription of toxin-antitoxin loci resulting in proteins to help bacteria survive better in these harsh conditions [9].

Like other Vibrios, *V*. *cholerae* harbors two circular chromosomes of 2.96MB (chromosome I) and 1.1MB (chromosome II), encoding 2770 and 1115 open reading frames (ORFs), respectively [10]. Most essential functional genes required for viability and growth are found on chromosome I, while chromosome II contains a large number of hypothetical genes and unknown-function genes. Moreover, chromosome II carries a 125.3 Kbp large gene capture and excision system called superintegron (SI) [11,12]. Although many *V*. *cholerae* SI-encoded ORFs are proteins with unknown function, some genes are thought to be important for cell functions including ribosomal and essential metabolic proteins. Interestingly, all known *V*. *cholerae* TA systems are also located in the SI region.

*V*. *cholerae* superintegron contains at least thirteen predicted toxin antitoxin loci (TA) [13]. A recent report adds additional four TA pairs in this region [14]. TA pairs were first discovered on plasmids that ensure maintenance of the plasmid by post-segregational killing. In this system, antitoxin proteins are short-lived, and the long-lived toxin kills daughter cells that do not contain the antitoxin-harboring plasmid [15,16]. This differential lability of toxin and antitoxin proteins results in maintenance of plasmids containing the TA locus. Since this initial discovery, many bacteria have been found to have chromosomally encoded TA systems. There are five types of TA systems based on the molecular nature of the antitoxin, which can be either RNA or protein based [17]. These toxin and cognate antitoxin encoding genes are typically very small and located in the same operon. The antitoxin directly counteracts the toxin by forming a complex, thus neutralizing toxicity to the cell. Under nutritional or other stress, antitoxin proteins may be degraded by enzymes such as Lon protease, allowing the toxin to exert its toxic activity by inhibiting cellular processes such as translation and replication. Since the initial discovery of plasmid-borne TA loci contributing to plasmid maintenance, several TA families have been discovered. Based on the toxins’ amino acid sequence and three dimensional similarities, they have been grouped into several families. Among these families some have been well described *relBE* [18], *parDE* [19], *higBA* [20], *phd/doc* [21], *ccd* [22], *vapBC* [23] and others. TA systems are ubiquitous, but are not essential for cell growth. They are considered to play important roles in survival under stress conditions and regulate a variety of important physiological functions such as persistence, antibiotic resistance, biofilm formation, and chronic infection [24]. For example, in *E*.*coli*, a five-TA system deletion strain results in overexpression of YjgK, which has a temporal influence on biofilm formation, resulting in decreased biofilm formation at 8-hr and an increase at 24-hr [25]. Toxin-antitoxin systems have also been implicated in antibiotic tolerance with well described mechanisms. For example, in *E*.*coli* and *Salmonella*, *hipA* and *shpAB* were found to increase multidrug tolerance by promoting a higher percentage of cells to take on a persister dormancy phenotype [26,27]. Moreover, recent research uncovered the long overlooked role of TA system in regulation of bacterial virulence. One study compared isolates of extraintestinal pathogenicity *E*. *coli* (ExPEC) and found that a subset of type II TA loci likely play an important role in pathogenicity by providing a competitive advantage in niche environments during colonization. In one ExPEC isolate, CFT073, the TA systems YefM-YoeB and YbaJ-Hha were associated with bladder colonization, while a third TA system, PasTI, is critical for colonization in kidneys [28]. Likewise, in *Salmonella Typhimurium*, the TA system *sehAB* shows transient activation that plays a role in triggering virulence expression in the peroral mouse model. The authors conclude that *sehAB* provides this pathogen a virulence advantage to survive in host organs [29].

**Fig 1:**
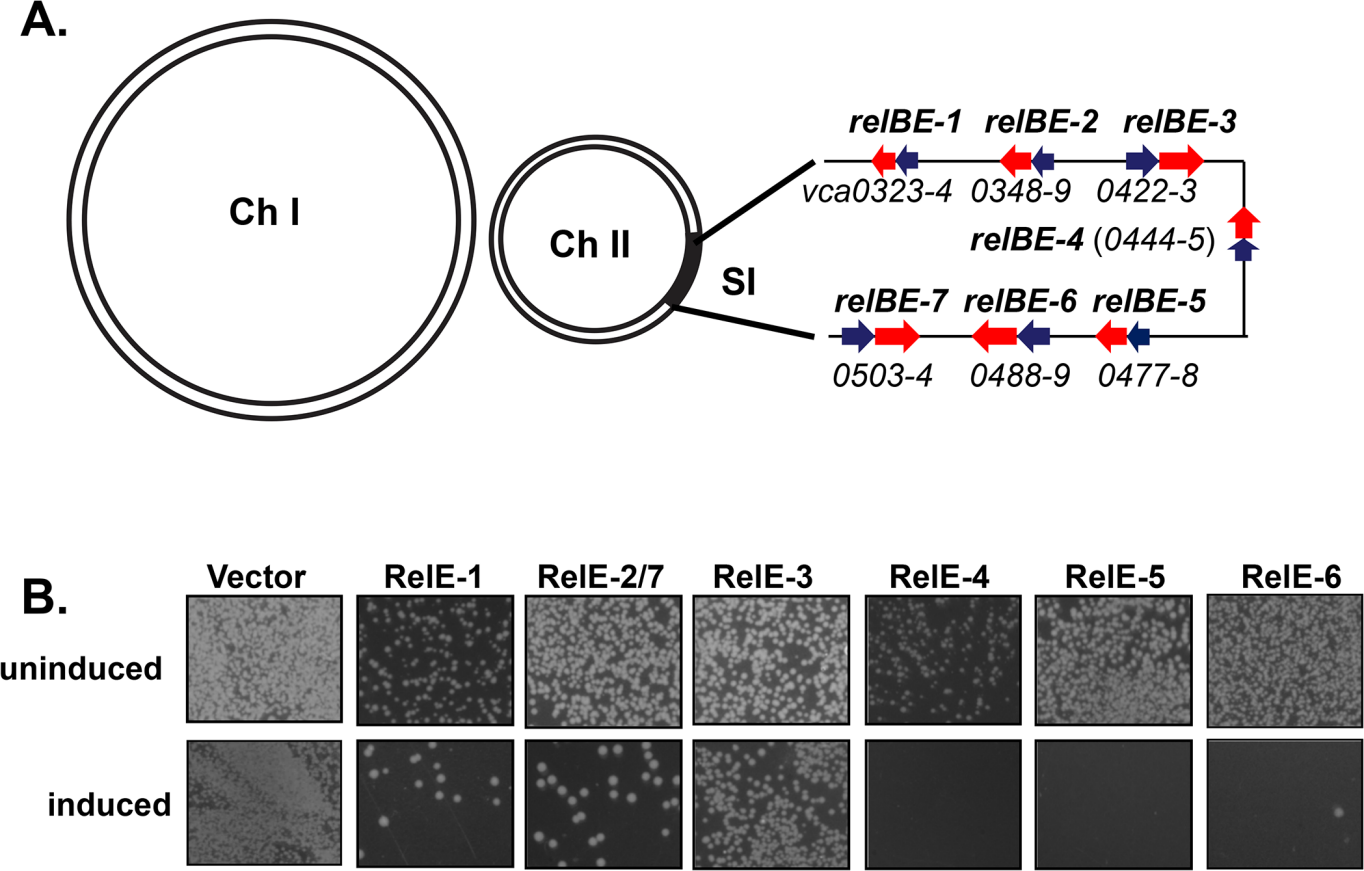
*V*. *cholerae* RelBE TA systems and their function. A. Location of RelBE-family in *Vibrio cholerae* chromosome II superintegron (SI). RelE toxins are labeled with red and RelB antitoxin blue. Genes are depicted in correct orientation with relative size, and in the absence of intervening non-TA genes. B. RelE toxin activity. Each of six *relE* genes was cloned into pBAD33. Purified construction plasmids were electroporated into BW27784 cells. After one hour recovery, same volume of culture was plated on LB agar with 0.2% glucose (uninduced) or 0.2% Arabinose (induced). Photographs were taken after 16h incubation at 37°C. pBAD33 vector was used as a control.

All *V*. *cholerae* TA loci belong to Classic Type II TA system in which the antitoxin is proteinaceous. BLASTP and TBLASTN assigns them to four families: *relBE*, *higBA*, *parDE* and *phd/doc* [13].They have similar regulatory modules in which the antitoxin is upstream of its cognate toxin, with only *higBA* exhibiting a reverse gene order [30]. The protein sequences of RelBE-2 and RelBE-7, ParDE-1 and ParDE-3 are identical. Of these TA pairs, *parDE*, *higBA* and *phd/doc* have been proven to be functional TA loci that encode bacteriostatic toxins, and it has been suggested that *parDE*, *higBA* contribute to the genetic stability of the SI [31–33]. For the RelBE family TA system, *relE* encodes an mRNA-cleaving toxin, which leads to growth inhibition of host cells. The *relB* gene encondes an antitoxin that neutralizes RelE toxicity [34]. Although it has been suggested that *V*. *cholerae* RelBE pairs are also involved in stabilizing the massive SI cassette array [14], no detailed deletion analysis of these pairs is available. Here we explore the physiological roles of the seven RelBE-family TA systems (Fig 1A). We constructed clean deletion strains of each RelBE-like toxin-antitoxin pair and examined the phenotypes of the mutants in mouse colonization, biofilm formation, antibiotic resistance, and growth under host-like stress conditions. Our results suggest that RelBE TA systems may fine-tune *V*. *cholerae* environmental survival and pathogenesis.

## Materials and Methods

### Ethical Statement

All animal experiments were done in accordance with NIH guidelines, the Animal Welfare Act and US federal law. All animal experiments were carried out in strict accordance with the animal protocol number 804529 that were approved by the IACUC Committee of the University of Pennsylvania. Total of 42 5-day-old infant CD-1 mice were used. All animals were housed in a centralized and AAALAC-accredited research animal facility that is fully staffed with trained husbandry, technical and veterinary personnel. As *V*. *cholerae* does not induce any symptoms in the animal model, the mice experience minimal discomforts. Method of euthanasia: Upon termination of the experiment, the infant mice will be euthanized by halothane inhalation followed by decapitation as is consistent with the recommendations of the panel on Euthanasia of the American Veterinary Medical Association.

### Strains, growth condition and medium

All strains were grown in Luria–Bertani (LB) broth supplemented with appropriate antibiotics and incubated at 37°C unless otherwise indicated. For virulence gene induction, AKI medium was used [35]. Plasmids and strains used in this study are listed in Table 1. For *V*. *cholerae*, strains are all derived from El Tor C6706 [36]. Concentrations of antibiotics used were: streptomycin 100 μg/mL, ampicillin 100 μg/mL, chloramphenicol 20 μg/mL for *E*.*coli* and 2 μg/mL for *V*. *cholerae*.

**Table 1:** Strains and plasmids used in this study.

Strain and plasmid	Genotype	Reference
*Vibrio cholerae*C6706	El Tor, Streptomycin resistance	[36]
*△relBE-1*	*lacZ* ^*-*^, *△vca0323vca0324* derivative of C6706	This study
*△relBE-2*	*lacZ* ^*-*^, *△vca0348vca0349* derivative of C6706	This study
*△relBE-3*	*lacZ* ^*-*^, *△vca0422vca0423* derivative of C6706	This study
*△relBE-4*	*lacZ* ^*-*^, *△vca0444vca0445* derivative of C6706	This study
*△relBE-5*	*lacZ* ^*-*^, *△vca0477vca0478* derivative of C6706	This study
*△relBE-6*	*lacZ* ^*-*^, *△vca0488vca0489* derivative of C6706	This study
*△relBE-7*	*lacZ* ^*-*^, *△vca0503vca0504* derivative of C6706	This study
*E*.*coli* DH5α*λpir*	F-,ø80d*lacZ*DM15,D(*lacZYA-argF*)U169, *deoR*, *recA*1, *endA*1, *hsdR*17(rk-, mk+),*phoA*, *supE*44, *thi*-1, *gyrA*96, *relA*1, *λ*pir	[48]
*E*.*coli*SM10*λpir*	*thi thr leu tonA lacY supE recA*::*RP4-2 Tc*::*Mu Km λ*pir	[49]
*E*.*coli* BW27784	* *Δ(*araD-araB*)*567* Δ(*araH-araF*)*570*(::*FRT*) Δ*ara Ep-532*::*FRTφP*_*cp18*_ *araE533*Δ(*rhaD-rhaB*)*568hsdR514* Δ*lacZ478*(::*rrnB-3*)	[40]
pWM91	R6K vector with a *sacB* gene, ampicillin resistance	[37]
pBAD33	Expression vector containing the pBAD promoter with a pACYC184 origin of replicon	[38]
pBBR-*luxCDABE*	Luminescence without promoter, chloramphenicol resistance	[39]
pWM91-*relBE-1*	Flanking regions of *relBE-1* in pWM91	This study
pWM91-*relBE-2*	Flanking regions of *relBE-2* in pWM91	This study
pWM91-*relBE-3*	Flanking regions of *relBE-3* in pWM91	This study
pWM91-*relBE-4*	Flanking regions of *relBE-4* in pWM91	This study
pWM91-*relBE-5*	Flanking regions of *relBE-5* in pWM91	This study
pWM91-*relBE-6*	Flanking regions of *relBE*-6 in pWM91	This study
pWM91-*relBE*-7	Flanking regions of *relBE-7* in pWM91	This study
pBAD33-*relE*-*1*	pBAD::*relE-1*,*relE-1* in *XbalI-PstI* sites of pBAD33	This study
pBAD33-*relE*-2/7	pBAD::*relE-2/7*,*relE-2/7* in *XbalI-PstI* sites of pBAD33	This study
pBAD33-*relE*-*3*	pBAD::*relE-3*,*relE-3* in *XbalI-HindⅢ* sites of pBAD33	This study
pBAD33-*relE*-*4*	pBAD::*relE-4*,*relE-4* in *XbalI-PstI* sites of pBAD33	This study
pBAD33-*relE*-*5*	pBAD::*relE-5*,*relE-5* in *XbalI-PstI* sites of pBAD33	This study
pBAD33-*relE*-*6*	pBAD::*relE-6*,*relE-6* in *XbalI-PstI* sites of pBAD33	This study
pP_*relBE-1*_ *-luxCDABE*	Promoter region of *relBE-1* in *SpeI* and *BamHI* sites of pBBR-*luxCDABE*	This study
pP_*relBE-2*_ *-luxCDABE*	Promoter region of *relBE*-*2* in *SpeI* and *BamHI* sites of pBBR-*luxCDABE*	This study
pP_*relBE-3*_ *-luxCDABE*	Promoter region of *relBE-3* in *SacI* and *BamHI* sites of pBBR-*luxCDABE*	This study
pP_*relBE-4*_ *-luxCDABE*	Promoter region of *relBE-4* in *SacI* and *BamHI* sites of pBBR-*luxCDABE*	This study
pP_*relBE-5*_ *-luxCDABE*	Promoter region of *relBE-5* in *SacI* and *BamHI* sites of pBBR-*luxCDABE*	This study
pP_*relBE-6*_ *-luxCDABE*	Promoter region of *relBE-6* in *SacI* and *BamHI* sites of pBBR-*luxCDABE*	This study
pP_*relBE-7*_ *-luxCDABE*	Promoter region of *relBE-7*in *SpeI* and *BamHI* sites of pBBR-*luxCDABE*	This study

### Constructions of in-frame deletions, overexpression constructs, and transcriptional reporters

In-frame deletions were constructed by cloning the regions flanking of the target genes into a suicide vector pWM91 containing a *sacB* counterselectable marker [37]. Double-crossover recombination mutants were selected using sucrose plates and confirmed by PCR. Five toxin-antitoxin system mutants were generated by scarless deletion of the entire opening reading frame containing both toxin and antitoxin genes. For toxin expression plasmids, each toxin gene was amplified by PCR from chromosomal DNA and was introduced into an arabinose induced expression plasmid pBAD33 [38]. Promoter region of each toxin-antitoxin operon was amplified by PCR and constructed into promoterless luminescence reporter plasmid pBBR-*luxCDABE* [39].

### Toxin activity assay

Each of six pBAD33-*relB* plasmids were transferred into *E*.*coli* BW27748[40] by electroporation. After a one hour recovery in LB liquid medium, equal amounts of cells were plated on LB agar containing glucose (0.2%) or arabinose (0. 2%) as well as appropriate antibiotics. Growth was monitored after the plates were incubated at 37°C overnight.

### Biofilm assays

Strains were grown overnight and inoculated 1:100 into in 10x75 mm borosilicate glass tubes containing 500 μl fresh LB medium and incubated at 22°C without shaking. After 24h and 48h incubation, biofilm quantification was performed by staining with crystal violet as described previously [41].

### Competition colonization assay

Competition assay followed infant mouse colonization protocol as described previously [42]. Each TA mutant (*lac*Z-) was mixed with wild type *V*.*cholerae* (*lac*Z+) at 1:1 ratio. Approximately 10^5^ cells were inoculated into 5 days old CD-1 infant mice. Infant mice were sacrificed after 20 hrs, when small intestines were collected, and homogenized. Output ratio of mutant to wild type was determined by plating serial diluted homogenate culture on LB agar plates containing X-gal (5-bromo-4-chloro-3-indolyl-beta-D-galacto-pyranoside). The competitive index was calculated by the output ratio of mutant to wild type dividing the input ratio of mutant to wild type.

### Measurement of TA mutant growth *in vitro*

All *V*. *cholerae* TA mutants were grown in LB liquid overnight and inoculated 1:1000 into LB liquid medium containing different stress signals as indicated. Measurement was performed in 96-well plates by a microplate reader (Biotek, Synergy|H1) at 37°C. For hydrogen peroxide H_2_O_2_ treatment, a disc diffusion assay was applied. Bacteria were normalized to OD_600_ = 1.0 (approximately10^9^CFU/mL), and then mixed 1:100 with 0.5% LB soft agar at 42°C and overlaid on LB agar plates. 10 μl of 5M H_2_O_2_ was spotted on 6mm diameter filter paper in the center of top layer. Diameter of inhibition zone was measured after 16-hr incubation at 37°C.

### Minimum inhibit concentration assays

Minimum inhibit concentration (MIC) was measured for each strain by using agar plate with increasing antibiotic gradients concentrations[43]. Each strain were streaked parallel to the axis of the antibiotic concentration gradient. After inoculation, plates were cultivated at 37°C for 16 hrs. MIC was determined by the minimum concentration to inhibit cell growth.

### Transcriptional analysis

All strains containing luminescence reporter plasmids were cultivated in LB medium overnight. 4 μl of each culture were inoculated into 4 mL AKI medium and LB medium respectively and then incubated at 37°C without shaking. Luminescence and OD_600_ were measured at the time points indicated using a Synergy H1 plate reader (Biotek).

## Results and Discussion

### Six of seven RelE homologues encode functional toxins that inhibit cell growth

As HigBA, ParDE, and Phd/Doc of Class II TA systems have been studied in *V*. *cholerae* [31–33], we investigate the roles of seven predicted *relBE* family loci. To do this, we first expressed each RelE toxin in *E*. *coli* to assess if any of these putative toxins inhibits bacterial growth. Each *relE* coding sequence was cloned into an arabinose-inducible expression plasmid (pBAD33) [38] as derivatives pBAD33-*relE* (as *relBE-2* and *relBE-7* have identical sequences, only one was examined). Derivate pBAD33-*relE* plasmids were electroporated into *E*.*coli* BW27784, in which a high-affinity arabinose transporter is constitutively expressed [40]. Growth of each culture harboring pBAD33-*relE* was monitored by plating cells on LB agar containing 0.2% glucose to repress the pBAD promotor or 0.2% arabinose to induce toxin production. Growth of *E*.*coli* containing *relE* overexpression plasmids was inhibited to different degrees when grown on arabinose-LB plates (Fig 1B, bottom panels) compared to glucose-LB plates (Fig 1B, top panels). This growth inhibition in the presence of RelE was nearly complete for *relE-4*, *5*, *6* demonstrating potent RelE toxicity to cells. Only RelE-3 did not result in discernable toxicity when expressed in *E*. *coli*, consistent with a previous report that RelE-3 contains a frameshift mutation [14]. These data suggest that at least six of seven *V*. *cholerae relE* TA loci encode functional toxins. These six *relB* genes likely encode functional antitoxin proteins to counteract their cognate toxins in *V*. *cholerae* under normal conditions. Other well studied *relE* toxins encode a global transcriptional inhibitor that inhibits cell growth by targeting mRNA and cleaving the coding region at ribosomal A site. Based on sequence homology, it is probable that *relE* toxins in *V*. *cholerae* act in a similar manner [34].

### RelBE systems affect maturation of biofilms

**Fig 2:**
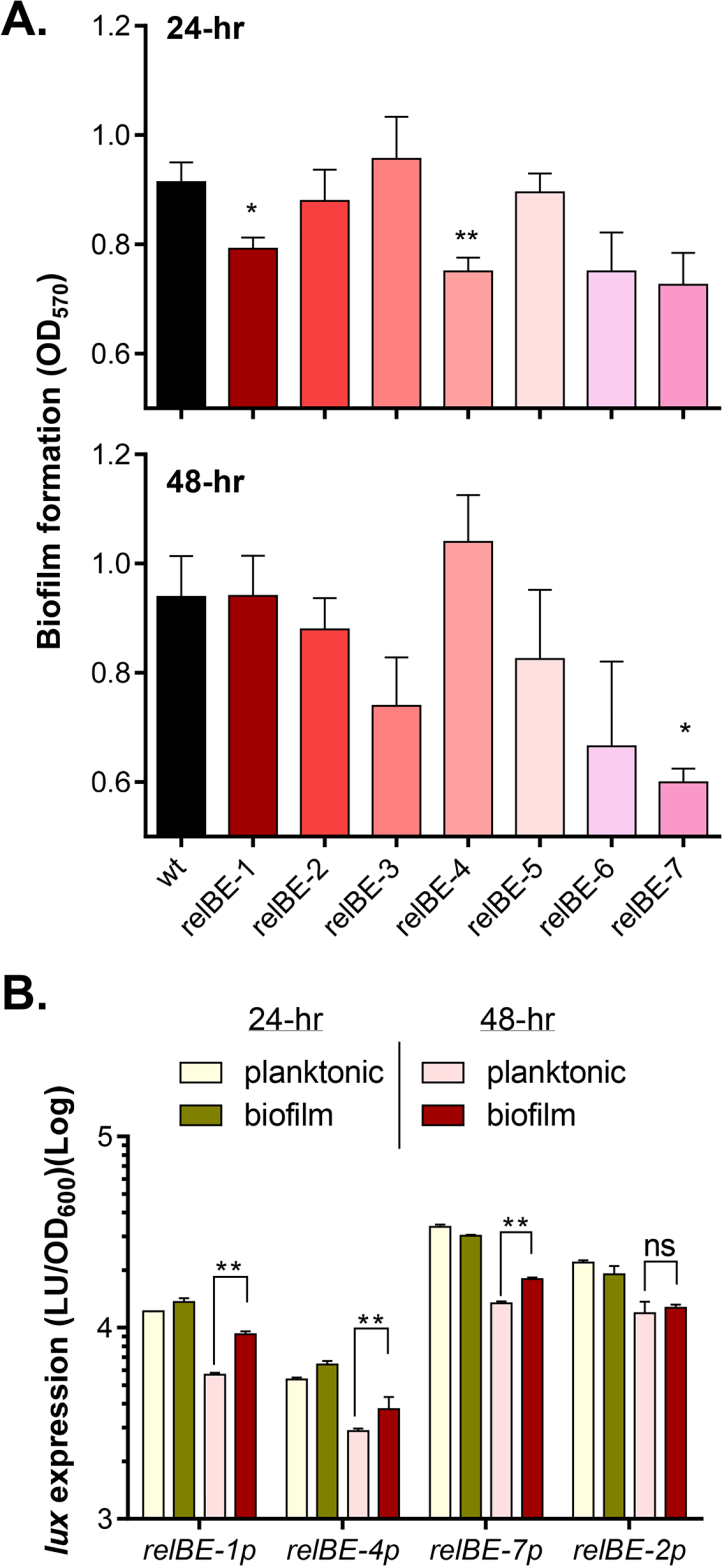
The effect of TA mutants in biofilm formation. **A.** Quantification of biofilms. Wild type and *relBE* mutants were inoculated 1:100 into LB and incubated at 22°C for 24 hrs (top panel) and 48 hrs (bottom panel). The biofilm mass was stained with crystal violet and quantified [50]. Data are mean and s.d. of three independent experiments. Student’s t-test P-values: *: P<0.05; **: P<0.01. **B.** Expression of indicated TA loci in planktonic cells and biofilm cells after 24 and 48 hours of growth. Wild type containing the promoter of each toxin-antitoxin operon fused to a luminescence reporter plasmid (*lux*) was inoculated into LB medium and cultivated without shaking at 22°C. Biofilms were washed with phosphate-buffered saline (PBS) three times and collected in LB medium. Luminescence was measured in planktonic and biofilm cells after 24-hr or 48-hr incubation. Data are mean and s.d. of three independent experiments. **: P<0.01. ns: no significance.

To investigate the physiological roles that RelBE TA systems may play in *V*. *cholerae*, we constructed deletions of each *relBE* locus. We then exposed these mutants to various conditions related to *V*. *cholerae* biology and compared the phenotype to wild type. We first examined whether deletion of any RelBE pairs affect *V*. *cholerae* biofilm formation. Each single RelBE TA loci mutant was successfully deleted without a growth defect under standard lab conditions. This suggested that none of these loci are crucial for cell viability. We found that at 24-hr, *relBE*-1and *relBE*-4 mutants formed significantly less biofilm than wild type. This defect was corrected by the 48 hour time point (Fig 2A), suggesting that biofilm formation may be delayed in these mutants. After 48, but not 24 hours of incubation, the *relBE-7* mutant also showed significantly decreased biofilm formation compared to wild type (Fig 2A), suggesting that RelBE-7 may be involved in biofilm maturation. Taken together, these data suggest that the RelBE family may contribute to a subtle but complex modulation of the biofilm developmental process, resulting in delayed or decreased biofilm formation in some cases.

Transcriptional expression of these three biofilm related toxin-antitoxin systems were determined via luminescence reporter plasmid in both in planktonic and biofilm cells. At 24-hr, expression of *relBE-1*, *-4*, and *-7* in planktonic and biofilm-associated cells was similar, but the level of the expression was nevertheless high compared to planktonic cells (Fig 2B), suggesting that *V*. *cholerae* produces these functional TA pairs at this stage of biofilm development. At 48-hr, all three TA were expressed more highly in biofilm-associated cells than in planktonic cells (Fig 2B). These results imply that these TA pairs may play certain roles specifically occurring in the biofilm-associated state. Interestingly, RelBE-2 has the same coding sequences as that of RelBE-7, but deletion of *relBE-2* showed little effects on biofilm formation (Fig 2A). It is possible that the expression between *relBE-2* and *relBE-7* is different. We found that compared to *relBE-7*, the expression of *relBE-2* was not induced in biofilm-associated cells (Fig 2B). These results may explain why *relBE-7*, but not *relBE-2*, is involved in biofilm maturation under the conditions tested.

### RelBE TA systems do not affect *V*. *cholerae* antibiotic resistance

We next examined whether these RelBE TA systems are involved in *V*. *cholerae* antibiotic resistance. The minimum inhibition concentration (MIC) was tested for various antibiotics in these single TA loci knockout mutants. Cultures of mutant and wild type *V*. *cholerae* were incubated into gradient plates with different antibiotics. Cell viability was measured across antibiotic concentrations, and the MIC calculated by the bacterial growth. The five antibiotics tested were ampicillin, chloramphenicol, gentamicin, tetracycline, and nalidixic acid (Table 2). We found that compared to wild type, none of these mutants displayed any significant difference on susceptibility of antibiotics tested. Although little difference in antibiotic resistance for individual TA mutants was detected, we cannot rule out that these *relBE* may cooperate with each other and contribute to resistance to antibiotics by working together.

**Table 2:** MIC for TA loci knockout mutants.

	Amp	Cm	Tc	Nal	Gen
wild type	3.55±0.17	0.90±0.17	0.48±0.06	0.57±0.06	1.10±0.11
*△relBE-1*	3.84±0.22	0.83±0.11	0.44±0.04	0.60±0.01	1.18±0.19
*△relBE-2*	3.60±0.26	0.81±0.01	0.48±0.01	0.56±0.04	1.10±0.03
*△relBE-3*	3.68±0.15	0.90±0.01	0.52±0.04	0.60±0.04	1.17±0.01
*△relBE-4*	3.70±0.13	0.81±0.22	0.50±0.03	0.56±0.02	1.02±0.05
*△relBE-5*	3.64±0.06	0.85±0.10	0.48±0.02	0.59±0.01	1.11±0.01
*△relBE-6*	3.80±0.15	0.84±0.10	0.48±0.01	0.52±0.04	1.15±0.13
*△relBE-7*	3.80±0.01	0.95±0.06	0.48±0.01	0.60±0.01	1.13±0.03

a. MIC is μg/ml (±s.d.). There is no significant difference between wild type and mutants.

### RelBE TA systems play limited role in *V*. *cholerae* host-like stress-resistance

**Fig 3:**
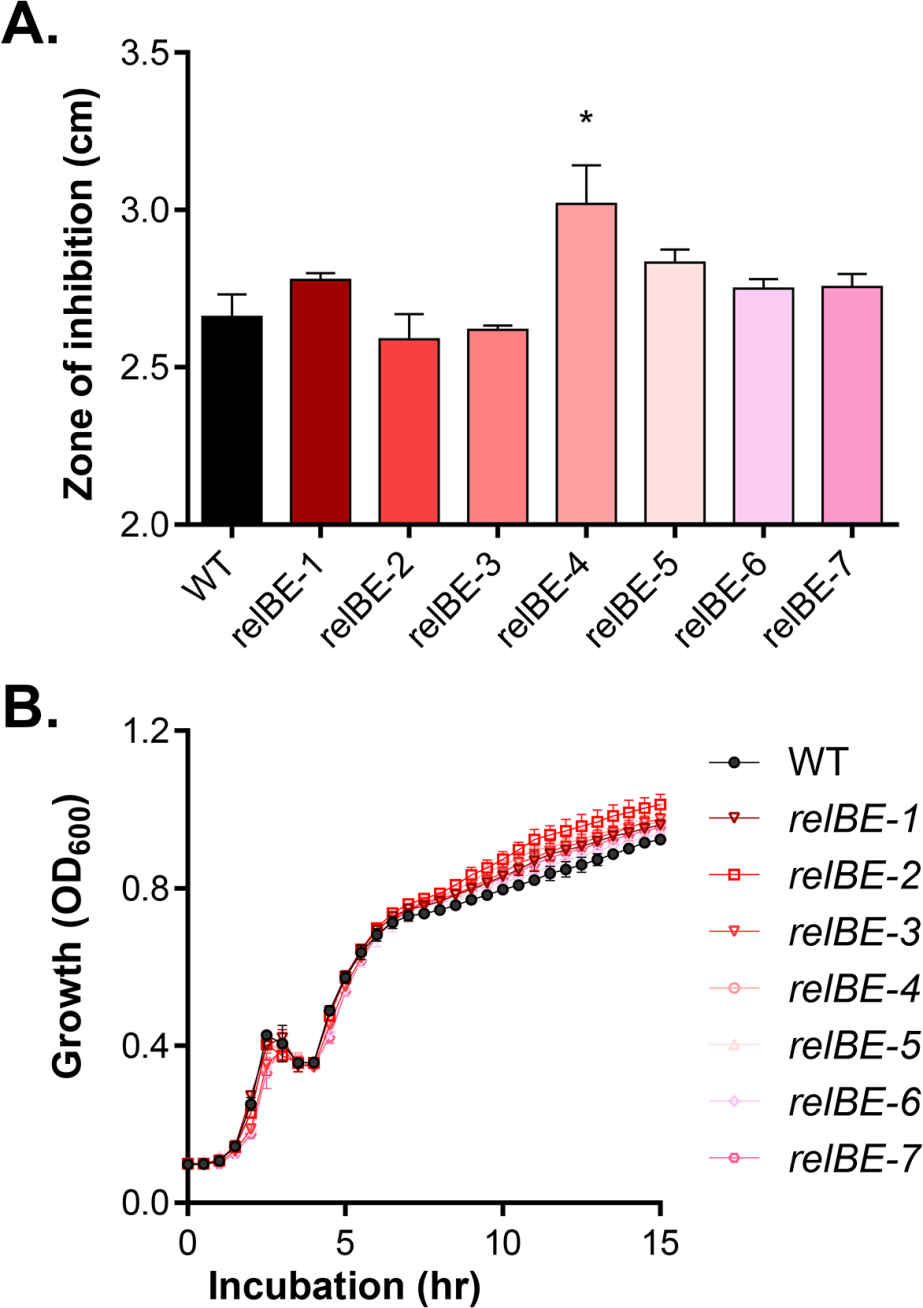
The effect of RelBE on *V*. *cholerae* resistance of stress. **A.** Disc assays for hydroperoxide resistance. Plates with indicated mutant in top-agar were incubated with a disc loaded with (5 M H_2_O_2_) at 37°C for 16 hrs. The diameter of inhibition zone was then measured. Data are mean and s.d. of three independent experiments. *: P<0.05. **B.** Bile resistance. Wild type and *relBE* mutants were inoculated 1:100 into LB containing 0.02% crude bile (Sigma, Co) and incubated at 37°C. OD_600_ was measured. Data are mean and s.d. of three independent experiments.

As *V*. *cholerae* may encounter a number of environmental stresses during infection, such as ROS and bile, we examined whether RelBE-type TA systems contribute to *V*. *cholerae* resistance to these insults. Using a disc assay, we examined the survival of each individual TA mutant in the presence of H_2_O_2_. We found that *relBE-4* mutant shows slightly enhanced sensitive to H_2_O_2_, but no other TA mutant had significantly different H_2_O_2_ sensitivity (Fig 3A). We also examined the effect of these TA systems on *V*. *cholerae* survival in the presence of bile or bile salts, the major component of bile. We grew wild type and *relBE* mutants in LB containing 0.2% crude porcine bile or 0.2% purified bile salt mix (Sigma, Co) at 37°C. No growth defect was detected in any of these mutants (Fig 3B and data not shown), suggesting that RelBE TA systems are not important for *V*. *cholerae* bile resistance.

### RelBE TA systems contribute to *V*. *cholerae* colonization

**Fig 4:**
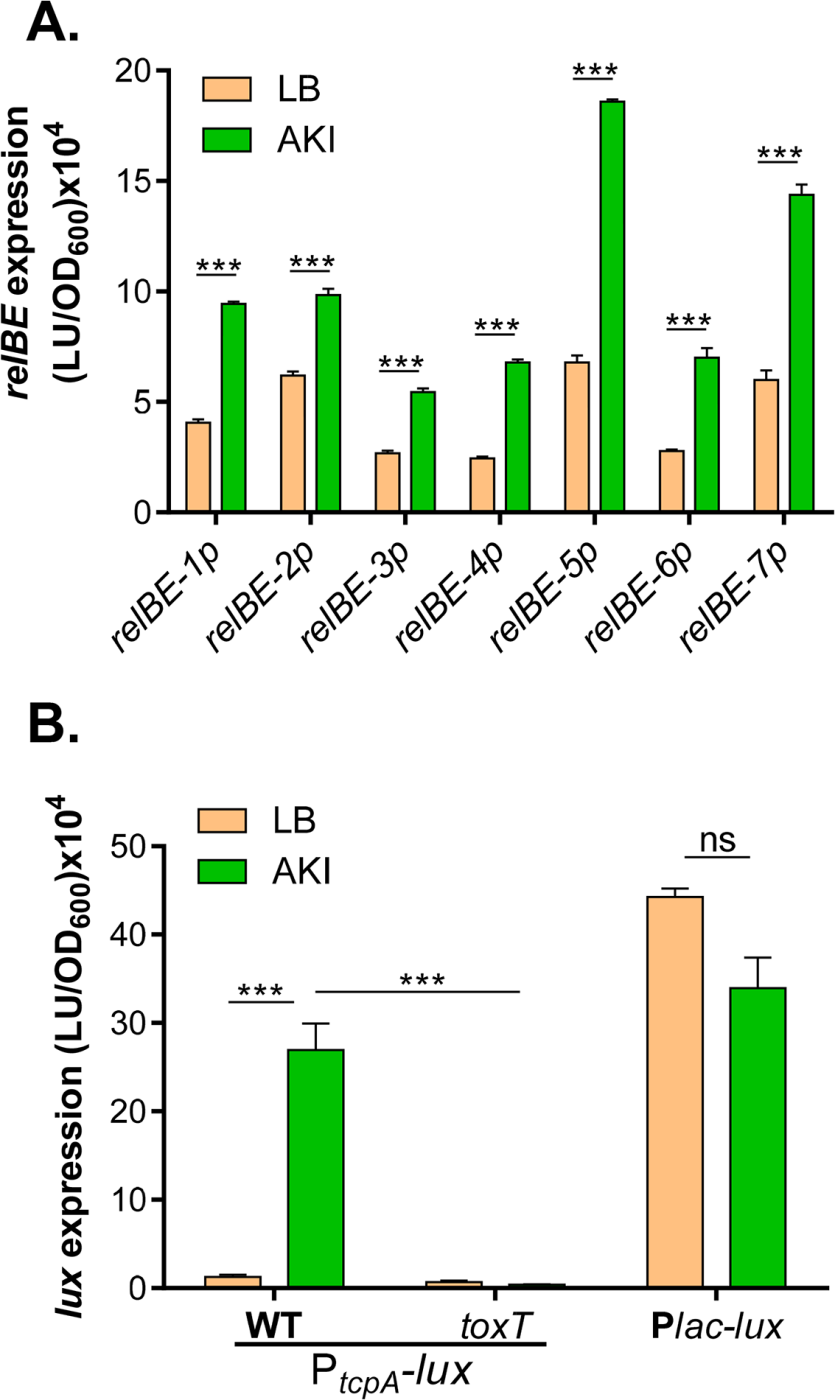
*relBE* expression under virulence inducing conditions. **A.** *relBE-lux*. Wild type strains containing each *relBE-luxCDABE* reporter plasmids were inoculated 1:1000 into LB or AKI medium and incubated at 37°C without shaking for 5 hrs. Luminescence was measured and normalized against OD_600_. Data are mean and s.d. of three independent experiments. ***: P<0.001. **B.** P_*tcpA*_ *-lux* and P_*lac-lux*_. Wild type or *toxT* mutant containing *tcpA-luxCDABE* reporter plasmids, or wild type containing P_*lac*_ *-luxCDABE* reporter plasmids were inoculated 1:1000 into LB or AKI medium and incubated at 37°C for 5 hrs. Luminescence was measured and normalized against OD_600_. Data are mean and s.d. of three independent experiments. ***: P<0.001. ns: no significance.

Next we investigated whether RelBE TA systems play any role in *V*. *cholerae* pathogenesis. We first examined whether these pairs are expressed under the *in vitro* virulence inducing condition. Wild type *V*. *cholerae* containing different *relBE-luxCDABE* fusion reporter plasmids were grown in AKI medium, which is known to induce all major virulence genes [35]. As a control, strains were also grown in LB medium. Intriguingly, we found that all seven *relBE* promoters were highly induced in AKI medium compared to LB (Fig 4A). To make sure that the induction of *relBE* genes in AKI is not an artifact, we used the same conditions to examine the expression of *tcpA*, the major virulence determinant, [44] in both wild type and *toxT* mutant, which is unable to express *tcpA* [45]. As expected, *tcpA* expression was highly induced in wild type but not in toxT mutant in AKI medium, not in LB (Fig 4B). In addition, we also examined a P_*lac*_-controlled constitutively expressed *luxCDABE* [39] in both LB and AKI medium and found that no significant difference was detected (Fig 4B). Taken together, these data suggest that all seven *relBE* TA loci are induced under *in vitro* virulence inducing conditions and may imply that they play a role in *V*. *cholerae* colonization or pathogenesis.

**Fig 5:**
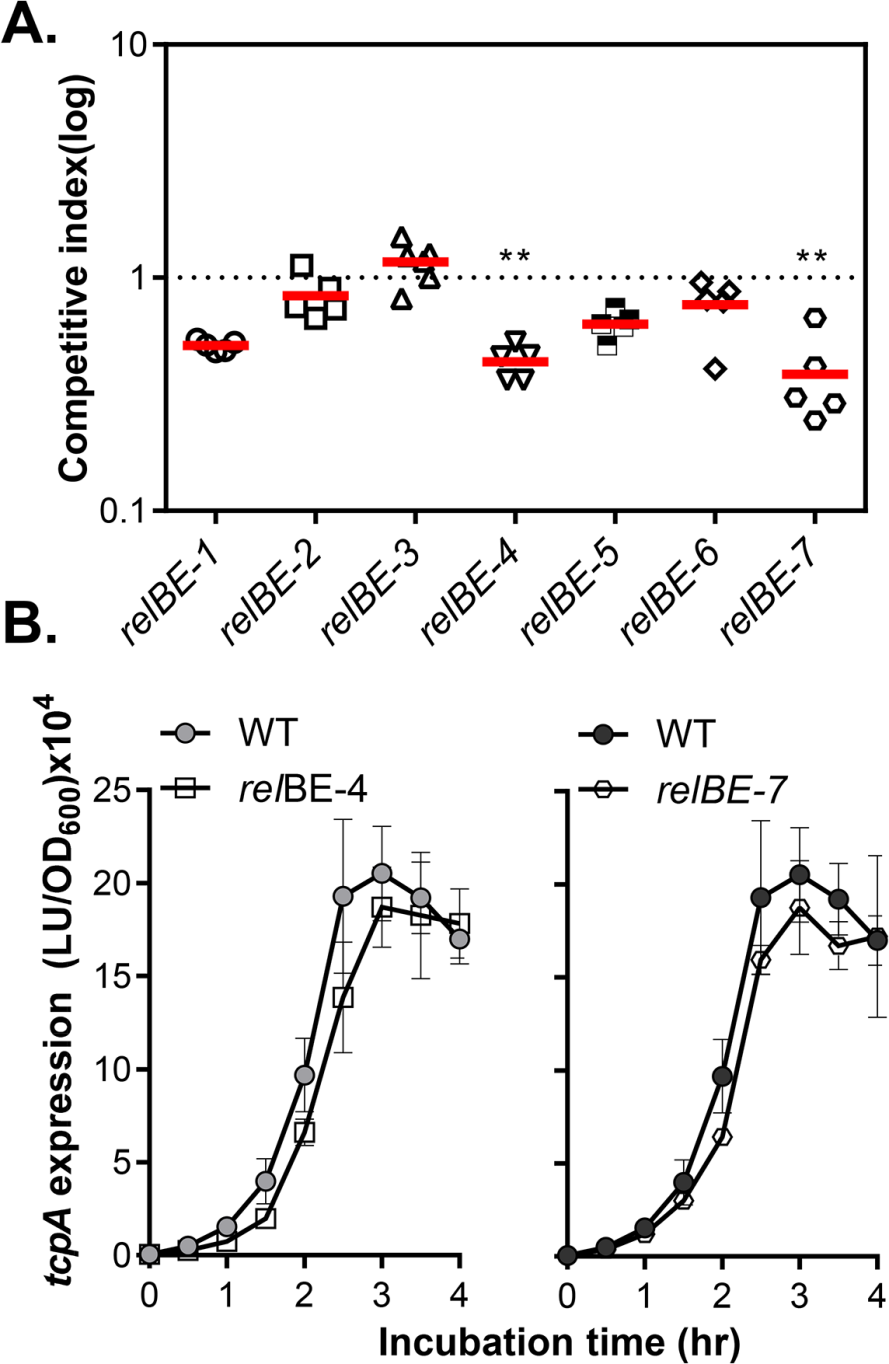
The effect of RelBE on virulence. **A.** Colonization competition assays. Approximately 10^5^ cells of wild type were mixed 1:1 with each *relBE* mutant and inoculated into 5-days–old infant mice. After 20-hr colonization, CFU of wild type and mutant was determined by plating of small intestinal homogenates. Horizontal lines denote median values for each group; n≥5 mice. **: P<0.01. **B.** *tcpA* expression. Wild type and mutants containing P_*tcpA*_-*luxCDABE* reporter plasmids were inoculated 1:1000 into AKI medium. The cultures were grown without shaking at 37°C. At the indicated time point, luminescence and OD_600_ were measured and normalized against OD_600_. Data are mean and s.d. of three independent experiments.

To address the possible involvement of TA systems in *V*. *cholerae* virulence, we used an infant mouse competition model [41] to test whether deletion in RelBE-family TA system affects colonization. For each mutant, equal amounts of wild type mutant were mixed at a 1:1 ratio and inoculated into 5-day-old infant mice. After 20 hours of colonization, mice were sacrificed, intestines harvested and homogenized, and homogenates were plated on LB-agar plates containing X-gal. Competitive index (CI) of each mutant was calculated as the output ratio of mutant to wild type normalized against the input ratio of mutant to wild type. Fig 5A shows that deletion of *relBE-4* and *relBE-7* impaired colonization (CI _*relBE-4*_ = 0.44,CI _*relBE-7*_ = 0.39, P<0.01), while other mutants were not attenuated. To explore the possible mechanism by which RelBE-4 and RelBE-7 modulate colonization, we examined if mutation of *relBE-4* and *relBE-7* ha any effect on virulence gene expression *in vitro*. We introduced a P_*tcpA*_-*luxCDABE* plasmid into these mutants and grew them in AKI inducing media. However, expression of *tcpA* did not differ from wild type for either of these two mutants (Fig 5B). These data suggest that the effect of RelBE on *V*. *cholerae* colonization may not be at the level of transcriptional regulation of virulence gene expression. Alternatively, virulence gene expression *in vitro* and *in vivo* may be different. Further studies are required to dissect the detailed molecular mechanisms of the effect of RelBE TA systems on *V*. *cholerae* colonization. Interestingly, although RelBE-2 and RelBE-7 share exactly the same protein sequences, the deletion mutants behaved differently during colonization (Fig 5A) and in biofilm formation (Fig 2A). This may be due to differential regulation of these two TA systems under different conditions. For example, when grown in LB medium, relBE-2 and relBE-7 were expressed at similar levels, but under the virulence inducing conditions, the induction of *relBE-7* was stronger than that of *relBE-2*, which may account for phenotypic differences (Fig 4A).

TA systems, small genetic modules consisting in general of 2 components, a stable toxin and its labile antitoxin, are prevalent in in prokaryotic bacterium [46]. The biological roles of different TA systems involves coping with stress by promoting programmed cell death or by inducing dormant cells, protection against DNA loss, such as increasing stability of superintegron, and prevention of invading DNA [46]. In this study, we examined the phenotypes of RelBE-family TA system single deletion mutants of *V*. *cholerae*. We found that the RelBE TA systems contribute to important traits of *V*. *cholerae* in terms of environmental survival and pathogenesis. It is possible that RelBE-family TA systems may play redundant roles in regulating biofilm formation as well as colonization. They may also cooperate with other TA systems. Future studies in which entire TA system family or all TA loci are deleted will allow us to uncover their biological functions in *V*. *cholerae*. Interestingly, like *V*. *cholerae*, many bacteria contain multiple homologous systems within a single genome. It has been shown that these systems do not cross-talk in general [47]. This may imply that each may play certain physiological roles that contribute to its persistence.
